# Preliminary In Vitro Evaluation of Vitamin E (α‐Tocopherol) Incorporation on Cytotoxicity, Antifungal Activity, and Surface Roughness of Denture Acrylic Soft Liner

**DOI:** 10.1155/ijod/9650820

**Published:** 2026-06-11

**Authors:** Neven S. Aref

**Affiliations:** ^1^ Department of Basic Oral Sciences and Dental Education, College of Dentistry, Qassim University, Buraydah, Saudi Arabia, qu.edu.sa; ^2^ Department of Dental Biomaterials, Faculty of Dentistry, Mansoura University, Mansoura, Egypt, mans.edu.eg

**Keywords:** α-tocopherol, acrylic soft liner, antifungal activity, cytotoxicity, denture biocompatibility, surface roughness, vitamin E

## Abstract

**Background:**

Denture soft liners are widely used in prosthodontics to improve patient comfort by cushioning the interface between the denture base and oral mucosa. However, clinical durability can be compromised by elevated surface roughness and adverse biological responses, including cytotoxicity and fungal colonization. Antioxidant supplementation—particularly with vitamin E (α‐tocopherol)—has been proposed as a strategy to enhance both the biocompatibility and surface quality of these materials.

**Materials and Methods:**

A commercially available chairside acrylic soft liner was modified with vitamin E at concentrations of 0.1, 0.2, and 0.5 wt.%, alongside an unmodified control group. Cytotoxicity was evaluated using the MTT cell viability assay on Vero cells, antifungal activity against *Candida albicans* was assessed through the agar diffusion method, and surface roughness was quantified with a calibrated profilometer. All data were analyzed using one‐way ANOVA followed by Tukey’s post‐hoc test at *p* ≤ 0.05.

**Results:**

Vitamin E incorporation produced a statistically significant, concentration‐dependent effect on both cell viability and surface roughness. The 0.2 wt.% group demonstrated the highest cell viability (48.0 ± 6.1%) and the lowest surface roughness (2.80 ± 0.23 µm) among all groups. Cell viability declined at 0.5 wt.%, yet remained significantly higher than the unmodified control. No antifungal inhibition zones were detected in any vitamin E‐modified group.

**Conclusions:**

Vitamin E incorporation improved selected biological and surface characteristics of the tested acrylic denture soft liner in a concentration‐dependent manner. The 0.2 wt.% formulation demonstrated the most favorable in vitro performance among the tested groups. However, these findings are preliminary, and comprehensive mechanical and physical evaluation is required before any clinical relevance or application can be established.

## 1. Introduction

In contemporary prosthodontic practice, denture soft liners serve a critical therapeutic function: they act as resilient, shock‐absorbing interfaces between the rigid acrylic denture base and the delicate oral mucosa, alleviating pressure points and improving patient comfort, particularly in individuals with atrophic or irregularly contoured ridges [1–5]. Despite these well‐established benefits, the long‐term clinical performance of soft liners is frequently limited by a combination of surface degradation, biological incompatibility, and susceptibility to microbial colonization.

A well‐documented concern associated with acrylic‐based soft liners is the leaching of residual monomers, plasticizers, and other low‐molecular‐weight constituents into the adjacent oral environment. These eluted compounds have been implicated in cytotoxic effects and unfavorable mucosal tissue responses, making rigorous biocompatibility assessment an essential prerequisite before clinical application [6–9]. Cytotoxicity testing, especially through standardized in vitro assays, is recognized as the foundational step in the biological evaluation of dental materials, enabling researchers to characterize cellular toxicity profiles before costly and time‐consuming clinical trials are undertaken [10, 11].

Beyond cytotoxicity, denture soft liners are particularly prone to fungal colonization, most notably by *Candida albicans*, the principal causative organism of denture stomatitis. The viscoelastic texture and inherent surface porosity of soft liners create a favorable environment for fungal adhesion and biofilm development, which, in turn, can perpetuate chronic mucosal inflammation and compromise denture hygiene [12, 13]. Surface roughness plays a central mediating role in this process: even modest increases in surface irregularity beyond a critical threshold (Ra > 0.2 µm) have been shown to promote microbial retention, accelerate plaque accumulation, and heighten the risk of inflammatory tissue reactions in denture wearers [14, 15].

The incorporation of antioxidant compounds into dental materials has attracted growing scientific interest in recent years. Antioxidants represent a chemically diverse class of molecules that protect cells and tissues from oxidative damage by neutralizing free radicals and maintaining redox homeostasis. Within dentistry, oxidative stress has been implicated in the pathogenesis of periodontal disease, dental caries, oral cancer, and mucosal inflammatory lesions. Consequently, antioxidant‐functionalized dental materials are being explored as a potential avenue for simultaneously improving biological performance and reducing tissue toxicity [16, 17]. Compounds such as vitamin C and E and plant‐derived polyphenols have demonstrated the capacity to suppress pro‐inflammatory mediators, reduce lipid peroxidation, and modulate cellular responses to biomaterial stimuli in oral tissues [17, 18].

Vitamin E, specifically α‐tocopherol, its most biologically active isoform, is a lipid‐soluble antioxidant with a well‐characterized mechanism of action involving the quenching of peroxyl radicals and the inhibition of lipid peroxidation chain reactions. In dental research, vitamin E has been investigated in the context of enamel erosion protection [19], restoration of bond strength following bleaching [20], and enhancement of oral wound healing [21]. Its oily, miscible nature makes it particularly amenable to direct incorporation into liquid‐phase components of acrylic systems without the need for additional carriers or encapsulation matrices.

Despite this promising background, the body of literature addressing vitamin E integration into denture soft liners remains limited, particularly regarding its potential influence on selected biological and surface characteristics. In addition to such properties, comprehensive assessment of denture liners also requires evaluation of mechanical and physical behaviors. However, the preliminary investigation of biological compatibility and surface performance represents an important first step in material screening and optimization.

This knowledge gap motivated the present preliminary in vitro investigation, which was designed to evaluate the effect of three vitamin E concentrations (0.1, 0.2, and 0.5 wt.%) on selected biological outcomes (cytotoxicity and antifungal response) and surface roughness of a commercially available chairside soft liner material.

The null hypothesis stated that vitamin E incorporation would produce no measurable effect on the evaluated biological parameters or surface roughness of the tested soft denture liner.

## 2. Materials and Methods

### 2.1. Study Design and Ethical Considerations

This study followed a preliminary in vitro experimental design focused on the evaluation of selected biological outcomes and surface roughness of a vitamin E‐modified chairside soft liner material. No human participants or live animals were involved; all cytotoxicity testing was performed using established, commercially sourced Vero cell lines. Accordingly, formal ethical approval was not required. The study was designed and reported in compliance with the CRIS guidelines for in vitro experimental research.

The study scope was limited to predefined biological and surface parameters as an initial in vitro assessment; comprehensive mechanical and long‐term performance testing were reserved for future investigations.

### 2.2. Materials

A commercially available chairside denture acrylic soft liner (Trusoft, Bosworth Co., Midland, TX, USA) served as the base material. Vitamin E in its oily form (α‐tocopherol) was obtained from Jamjoom Pharmaceuticals Co. (Jeddah, Saudi Arabia). Vitamin E was added directly to the liquid component of the soft liner at three concentrations: 0.1, 0.2, and 0.5 wt. %. Mixtures were placed on a magnetic stirrer for 24 h to ensure complete homogeneity before specimen fabrication. Four experimental groups were defined:

Group I (Control): unmodified soft liner
Group II: 0.1 wt.% vitamin E‐modified soft liner
Group III: 0.2 wt.% vitamin E‐modified soft liner
Group IV: 0.5 wt.% vitamin E‐modified soft liner.

The concentration range (0.1–0.5 wt. %) was deliberately selected to encompass low‐to‐moderate incorporation levels, thereby balancing potential biocompatibility enhancement against the risk of adverse effects on the material’s physicochemical integrity. Higher concentrations were intentionally excluded to prevent polymer network destabilization. Accordingly, the present study was restricted to selected biological and surface evaluations as an initial screening phase of material assessment.

### 2.3. Specimen Preparation

A total of 120 disc‐shaped specimens (40 per test parameter, 10 per group) were fabricated by mixing the liquid and powder components of the different experimental groups according to the manufacturer’s instructions. The mixture was poured into a stainless‐steel mold supported by a glass slab, covered with a second glass slab, and maintained under controlled pressure until complete curing. Specimens were carefully demoulded, edges were trimmed and smoothed, and all specimens were stored in distilled water at 37°C for 48 h prior to testing. All measurements were performed across replicate specimens within each group to maximize the reliability.

### 2.4. Cytotoxicity Assessment (MTT Assay)

Disc‐shaped specimens (1 mm thick and 10 mm diameter) were prepared for cytotoxicity evaluation. Prior to testing, specimens were cleaned with distilled water for 20 min and exposed to ultraviolet irradiation for an additional 20 min to eliminate fabrication contaminants. Vero cells (African green monkey kidney epithelial cells) were cultured in a complete growth medium in sterile, gamma‐irradiated polystyrene plates. Cells were harvested by trypsinization (0.25% trypsin, 37°C, 5 min), resuspended in fresh growth medium, and counted using the Trypan blue exclusion method and a hemocytometer. The cell suspension was adjusted to 30 × 10^5^ cells/mL and dispensed into 6‐well plates (2 mL/well). Specimen discs were introduced 24 h after cell seeding to allow for full cell attachment and equilibration. Cell viability was quantified using the standard MTT colorimetric assay, and the results were expressed as percentages relative to the unmodified control group.

### 2.5. Antifungal Activity Testing

The antifungal activity was evaluated by the agar diffusion method. Disc specimens (8 mm diameter and 2 mm thickness) were prepared for each group. The *Candida albicans* strain was sourced from the Microbiology and Immunology Department, Faculty of Pharmacy, Mansoura University, Egypt, and cultured on Sabouraud dextrose agar overnight at 37°C. Petri dishes (100 mm diameter) were prepared with 15 mL of molten agar inoculated with 100 µL of the fungal suspension (pH 7.5). Specimens were carefully positioned on the solidified agar surface, and plates were incubated aerobically at 37°C for 48 h. Fluconazole‐impregnated cellulose discs (8 mm, 5 µg/disc) served as positive controls. Zones of inhibition were measured in millimeters at three equidistant points around each specimen, and the mean value was recorded as the antifungal outcome.

### 2.6. Surface Roughness Measurement

Surface roughness specimens (10 mm diameter and 2 mm thickness) were prepared using standardized stainless‐steel molds. Surface topography was quantified at five measurement points per specimen, positioned at least 0.5 mm apart and 1.0 mm from the disc periphery. A calibrated contact profilometer with a diamond stylus tip (radius: 5 µm) was employed, with a measurement speed of 0.5 mm/s, an applied force of 4 mN, and a cutoff wavelength of 0.8 mm over a 3.0 mm stylus traverse. Average roughness values (Ra, µm) were calculated by averaging all five‐point measurements per specimen.

### 2.7. Sample Size Calculation

Sample size determination was based on a one‐way ANOVA model with four independent groups, employing a significance level (α) of 0.05, a statistical power of 80%, and a medium‐to‐large effect size (*f* = 0.40). The calculated minimum required sample size was 10 specimens per group, which was adopted in this study to increase measurement reliability and account for experimental variability.

### 2.8. Statistical Analysis

All statistical analyses were performed using SPSS software (Version 25.0; IBM Corp., Armonk, NY, USA). Data normality was verified using the Shapiro–Wilk test. As all datasets satisfied the normality assumption, one‐way ANOVA was applied for intergroup comparisons, followed by Tukey’s post‐hoc test to identify specific pairwise differences. Statistical significance was defined at *p* ≤ 0.05 for all comparisons.

## 3. Results

**Figure 1:**
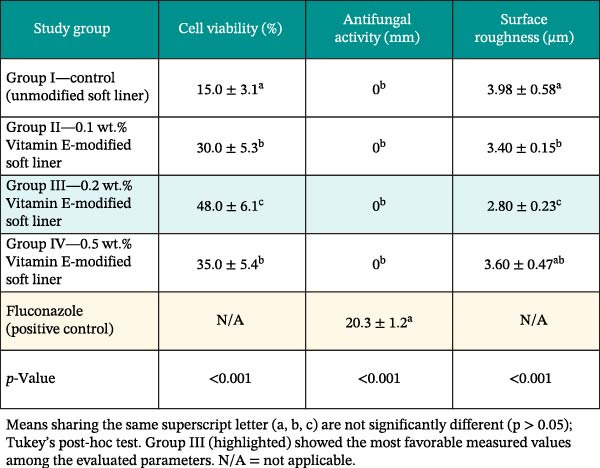
Descriptive statistics, one‐way ANOVA, and post‐hoc Tukey test results for all evaluated parameters.

**Figure 2:**
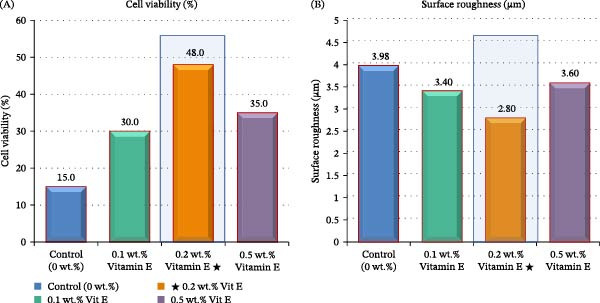
Bar chart comparing (A) cell viability (%) and (B) surface roughness (µm) across experimental groups. Group III (0.2 wt.% vitamin E) showed comparatively favorable values, with the highest cell viability and lowest surface roughness values (highlighted region). 0.2 wt.% group: highest cell viability (48.0 ± 6.1%) and lowest surface roughness (2.80 ± 0.23 μm). Mean ± SD; *n* = 10. One‐way ANOVA + Tukey HSD; *p* < 0.001.

The results of the evaluated biological parameters and surface roughness are summarized in Figure 1, with graphical representation provided in Figure 2.

### 3.1. Cytotoxicity (Cell Viability)

**Figure 3:**
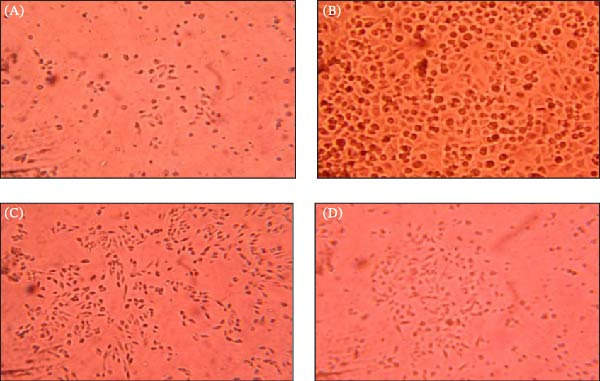
Micrographs of the cell viability (%) of the experimental groups; (A): Control (unmodified soft liner), (B): 0.1 wt.% vitamin E‐modified soft liner, (C): 0.2 wt.% vitamin E‐modified soft liner, and (D): 0.5 wt.% vitamin E‐modified soft liner.

One‐way ANOVA revealed a statistically significant difference in cell viability among the experimental groups (*p*  < 0.001). The unmodified control group (Group I) exhibited the lowest cell viability of all groups (15.0 ± 3.1%), consistent with residual monomer‐mediated cytotoxicity. All vitamin E‐modified groups showed significantly higher cell viability than the control (*p*  < 0.05). Group II (0.1 wt.%) demonstrated a moderate improvement (30.0 ± 5.3%), while Group III (0.2 wt.%) revealed the highest cell viability across all experimental groups (48.0 ± 6.1%). Increasing the concentration to 0.5 wt.% (Group IV) resulted in a statistically significant decline in cell viability (35.0 ± 5.4%) compared to Group III (*p*  < 0.05), although it remained significantly superior to the control. No significant difference was observed between Groups II and IV (*p*  > 0.05). Representative cell micrographs for all groups are shown in Figure 3.

### 3.2. Antifungal Activity

No inhibition zones were observed in any vitamin E‐modified group or in the unmodified control; all groups recorded zones of 0 mm. The only statistically significant difference detected was between the experimental groups and the fluconazole positive control, which produced a mean inhibition zone of 20.3 ± 1.2 mm (*p*  < 0.001). These findings confirm the absence of intrinsic antifungal activity in all tested soft liner formulations, regardless of vitamin E concentration.

### 3.3. Surface Roughness

One‐way ANOVA demonstrated a statistically significant difference in surface roughness among the groups (*p*  < 0.001). The control group exhibited the highest mean Ra value (3.98 ± 0.58 µm). Group III (0.2 wt.% vitamin E) demonstrated the lowest measured surface roughness (2.80 ± 0.23 µm), differing significantly from all other groups (*p*  < 0.05). Groups II (3.40 ± 0.15 µm) and IV (3.60 ± 0.47 µm) showed intermediate values; no statistically significant difference was found between Group IV and either the control or Group II (*p*  > 0.05).

## 4. Discussion

The principal objective of this preliminary study was to evaluate how vitamin E (α‐tocopherol) at three incremental concentrations may influence the selected biological and surface properties of a chairside acrylic denture soft liner. The null hypothesis was partially rejected: vitamin E incorporation produced statistically significant changes in cell viability and surface roughness but no detectable antifungal activity against *Candida albicans*.

### 4.1. Cytotoxicity and Cell Viability

The markedly low cell viability observed in the unmodified control group (15.0%) reflects the well‐recognized cytotoxicity associated with residual methyl methacrylate (MMA) monomers released from acrylic polymers. These small, lipophilic molecules can compromise cell membrane integrity, trigger oxidative DNA damage, and induce cytolysis at elevated concentrations, effects that may be partially attenuated in vivo by salivary dilution and binding but remain significant in in vitro settings [22].

The significant improvement in cell viability at 0.1 and 0.2 wt.% vitamin E is mechanistically consistent with the compound’s established role as a chain‐breaking antioxidant: by scavenging peroxyl radicals and inhibiting lipid peroxidation, α‐tocopherol reduces the downstream oxidative burden imposed on cells by polymer‐derived leachates [23, 24]. This aligns with a growing body of literature demonstrating that residual resin monomers generate reactive oxygen species (ROS), deplete intracellular glutathione reserves, impair mitochondrial function, and activate apoptotic cascades, all processes that antioxidant supplementation can meaningfully attenuate [25–28].

The observed reduction in cell viability at 0.5 wt. % compared to 0.2 wt. %, however, introduces an important nuance. Excessive antioxidant loading may interfere with free‐radical‐mediated polymerization kinetics, leading to incomplete cross‐linking [28] and consequently higher residual monomer concentrations—consistent with established reports that increased leachable components from insufficiently polymerized resin systems contribute to paradoxically elevated cytotoxicity at supraoptimal additive concentrations [29]. This concentration‐dependent inflection point underscores the importance of optimizing additive dosage: the biological response is not linearly proportional to antioxidant content, and there exists a narrow optimal concentration range, in this case, centered at 0.2 wt.% beyond which benefits diminish. This finding is consistent with reports demonstrating that antioxidant supplementation can meaningfully attenuate monomer‐induced cytotoxicity within a defined concentration window and that exceeding the optimal dose may paradoxically compromise rather than enhance cellular protection [30, 31].

The present findings are broadly consistent with published studies demonstrating that antioxidant‐modified polymeric dental materials exhibit improved cytocompatibility through reduced membrane disruption and enhanced polymer stability [32, 33]. However, the material‐specific nature of the optimal concentration observed here highlights the need for individual evaluation of each antioxidant–material combination rather than reliance on generalized concentration guidelines.

### 4.2. Antifungal Activity

The complete absence of antifungal inhibition zones across all vitamin E‐modified groups, in contrast to the robust activity of the fluconazole positive control (20.3 ± 1.2 mm), indicates that vitamin E does not provide direct antifungal efficacy against *Candida albicans* when incorporated into a solid polymeric matrix at the concentrations tested. This finding is consistent with a prior report noting that vitamin E does not provide direct antifungal efficacy against Candida species and may even reduce the antifungal activity of co‐administered agents [34].

Interestingly, vitamin E combined with natural compounds such as lycopene has demonstrated enhanced antimicrobial activity against oral pathogens including Candida albicans [35], and vitamin E has also been shown to enhance bacterial killing when combined with conventional antibiotics through inhibition of bacterial lipocalin binding [36], suggesting that combination strategies may offer broader antimicrobial potential than vitamin E alone. The contrast between vitamin E and other natural antioxidants such as curcumin, which has demonstrated measureable antifungal activity when integrated into denture liner materials [37], further suggests that antifungal effects are highly compound‐specific and may depend on mechanisms beyond oxidative stress modulation [38]. These observations collectively imply that achieving adequate antifungal protection in soft liner materials will likely require the co‐incorporation of dedicated antifungal agents alongside antioxidants rather than reliance on a single compound for dual functionality. Indeed, systematic review evidence confirms that dedicated antifungal agents—including fluconazole—when directly incorporated into soft liner matrices produce consistent and clinically meaningful inhibitory activity against *Candida albicans*, underscoring the need for purpose‐designed antifungal strategies rather than reliance on antioxidant compounds alone for this purpose [39, 40].

### 4.3. Surface Roughness

The significant reduction in surface roughness at 0.2 wt.% vitamin E (Ra = 2.80 µm) relative to the control (Ra = 3.98 µm) may represent a favorable material‐related finding under the present in vitro conditions. Surface roughness is a well‐established determinant of denture plaque formation, microbial adhesion, and the risk of denture stomatitis: Ra values above the critical threshold of ~0.2 µm facilitate bacterial and fungal attachment, while surfaces with elevated roughness are associated with chronic mucosal inflammation in denture wearers [14, 41]. Although all groups in this study exhibited Ra values above this theoretical clinical threshold, likely reflecting the intrinsic softness and surface porosity of the material, the relative reduction at 0.2 wt.% represents a relative improvement in measured surface quality.

The plasticizing effect of vitamin E′s oily nature, previously documented in polymeric film systems [42], may similarly underlie the observed surface improvements at moderate concentrations in the present study. By enhancing polymer chain mobility and promoting more uniform material flow during specimen setting, α‐tocopherol may reduce microstructural surface defects and improve overall surface homogeneity—consistent with observations of oily additive incorporation in other dental material systems [43]. Conversely, the increase in surface roughness at 0.5 wt.% is consistent with phase separation or microscale aggregation of excess vitamin E within the polymer matrix, a phenomenon also reported for other oily additives at supraoptimal loading [44]. This biphasic surface response mirrors the cell viability trend, reinforcing the concept of a narrow optimal concentration for vitamin E in this material system.

### 4.4. Potential Implications and Future Perspectives

The present findings suggest that vitamin E incorporation may contribute to improved biological response and reduced surface roughness of the tested chairside soft liner under controlled in vitro conditions. Such observations may be relevant for tissue‐contacting interim liner materials, particularly where minimizing additional irritation is desirable. However, these results should be interpreted cautiously as clinical suitability cannot be inferred from the present data alone. Comprehensive evaluation of mechanical behavior, bond durability, aging resistance, and long‐term in vivo performance remains necessary before any clinical recommendation can be made. In addition, combination strategies involving antioxidants together with dedicated antifungal agents may warrant further investigation.

Accordingly, the current findings are best regarded as foundational evidence to guide subsequent material optimization and comprehensive functional testing.

## 5. Limitations of the Study

This study was conducted entirely under in vitro conditions, which inherently cannot replicate the dynamic complexity of the oral environment. Variables such as thermocycling, salivary protein adsorption, enzymatic degradation, and occlusal loading forces, all of which influence the long‐term performance of soft liner materials, were not included in the present experimental design. Furthermore, the Vero cell line used for cytotoxicity assessment is derived from nonoral tissue (monkey kidney epithelium) and may not fully represent the biological responses of human oral mucosal cells. Future studies should incorporate primary oral epithelial or fibroblast cell cultures to improve the translational relevance.

Additionally, the present study did not include comprehensive mechanical and physical characterization, such as hardness, tear resistance, bond durability, viscoelastic behavior, or fatigue performance, which are relevant to functional clinical service. Therefore, the current findings should be interpreted as preliminary and limited to the evaluated biological and surface parameters. Future investigations should integrate mechanical testing, artificial aging protocols, and clinical assessment to establish the overall material suitability.

## 6. Conclusions

Within the limitations of this preliminary in vitro study, incorporation of vitamin E (α‐tocopherol) into a chairside acrylic denture soft liner produced concentration‐dependent improvements in the selected biological response and surface roughness. Among the tested formulations, 0.2 wt. % demonstrated the most favorable overall in vitro performance. No antifungal activity against *Candida albicans* was observed at any tested concentration. These findings provide initial evidence for further material development; however, comprehensive mechanical, physical, aging, and clinical evaluations remain necessary before clinical applicability can be determined.

## Author Contributions

**Neven S. Aref**: conceptualization, methodology, validation, formal analysis, investigation, resources, data curation, writing – original draft, writing – review and editing, visualization, project administration.

## Funding

The author received no specific funding for this work.

## Disclosure

All scientific content, data interpretation, and conclusions were generated exclusively by the author.

## Ethics Statement

This study is an in vitro experimental study. No human participants or live animals were involved. Commercially available Vero cell lines were used. Ethical approval was, therefore, not required.

## Consent

The author has nothing to report.

## Conflicts of Interest

The author declares no conflicts of interest.

## Data Availability

The data supporting the findings of this study are available from the corresponding author upon reasonable request.

## References

[1] Craig R. G. and Powers J. M. , Restorative Dental Materials, 2002, 11th edition, Mosby, St. Louis.

[2] Zarb G. A. , Bolender C. L. , Eckert S. E. , Jacob R. F. , Fenton A. H. , and Mericske-Stern R. , Prosthodontic Treatment for Edentulous Patients: Complete Dentures and Implant-Supported Prostheses, 2004, 12th edition, Mosby, St. Louis.

[3] Braden M. , Wright P. S. , and Parker S. , Soft Lining Materials—A Review, The European Journal of Prosthodontics and Restorative Dentistry. (1995) 3, no. 4, 163–174. 8601159

[4] Anusavice K. J. , Phillips’ Science of Dental Materials, 1996, 10th edition, WB Saunders, Philadelphia.

[5] Causton B. E. , O’Brien W. J. , Denture Base Polymers and Liners, Dental Materials and Their Selection, 1997, 2nd edition, Quintessence, Chicago, 90–92.

[6] Urban V. M. , Machado A. L. , Oliveira R. V. , Vergani C. E. , Pavarina A. C. , and Cass Q. B. , Residual Monomer of Reline Acrylic Resins: Effect of Water-Bath Post-Polymerization Treatment, Dental Materials. (2007) 23, no. 3, 363–368, 10.1016/j.dental.2006.01.021. 16620950

[7] Bettencourt A. F. , Neves C. B. , de Almeida M. S. , Pinheiro L. M. , Oliveira S. A. , and Lopes L. P. , Biodegradation of Acrylic-Based Resins: A Review, Dental Materials. (2010) 26, no. 5, e171–e180, 10.1016/j.dental.2010.01.006. 20189238

[8] Vallittu P. K. , Ruyter I. E. , and Buykuilmaz S. , Effect of Polymerization Temperature and Time on the Residual Monomer Content of Denture Base Polymers, European Journal of Oral Sciences. (1998) 25, no. 7, 560–565, 10.1046/j.1365-2842.1998.00263.x. 9527360

[9] Kawaguchi M. , Takahashi Y. , Fukushima T. , and Habu T. , Effect of Light-Exposure Duration on the Amount of Leachable Monomers From Light-Activated Reline Material, The Journal of Prosthetic Dentistry. (1996) 75, 183–187, 10.1016/S0022-3913(96)90097-9. 8667278

[10] International Organization for Standardization , Biological Evaluation of Medical Devices—Part 5: Tests for In Vitro Cytotoxicity, 2009, ISO, ISO 10993-5.

[11] Schmalz G. , Use of Cell Cultures for Toxicity Testing of Dental Materials—Advantages and Limitations, Journal of Dentistry. (1994) 22, no. suppl 2, S6–S11, 10.1016/0300-5712(94)90032-9. 7844275

[12] Radford D. R. , Challacombe S. J. , and Walter J. D. , Denture Plaque and Adherence of *Candida albicans* to Denture Base Materials In Vivo and In Vitro, Journal of Prosthetic Dentistry. (1999) 82, no. 4, 453–459. 10.1177/10454411990100010501 10759429

[13] Nikawa H. , Hamada T. , and Yamamoto T. , Denture Plaque—Past and Recent Concerns, Journal of Dentistry. (1998) 26, no. 4, 299–304, 10.1016/S0300-5712(97)00026-2. 9611934

[14] Bollenl C. M. L. , Lambrechts P. , and Quirynen M. , Comparison of Surface Roughness of Oral Hard Materials to the Threshold Surface Roughness for Bacterial Plaque Retention: A Review, Dental Materials. (1997) 13, no. 4, 258–269, 10.1016/S0109-5641(97)80038-3. 11696906

[15] Quirynen M. and Bollen C. M. L. , The Influence of Surface Roughness and Surface Free Energy on Supra- and Subgingival Plaque Formation in Man, Journal of Clinical Periodontology. (1995) 22, no. 1, 1–4, 10.1111/j.1600-051X.1995.tb01765.x. 7706534

[16] Parihar A. S. , Dutta B. , Yadav D. , Narayanan M. S. , Sahney T. , and Nandamuri S. , Exploring the Benefits of Antioxidants in Dentistry: New Frontiers in Oral Care, Journal of Pharmacy and Bioallied Sciences. (2024) 16, no. Suppl 3, S1926–S1928, 10.4103/jpbs.jpbs_6_24. 39346303 PMC11426609

[17] Qi F. , Huang H. , Wang M. , Rong W. , and Wang J. , Applications of Antioxidants in Dental Procedures, Antioxidants. (2022) 11, no. 12, 10.3390/antiox11122492, 2492. 36552699 PMC9774737

[18] Wang Y. , Andrukhov O. , and Rausch-Fan X. , Oxidative Stress and Antioxidant System in Periodontitis, Frontiers in Physiology. (2017) 8, 10.3389/fphys.2017.00910, 910. 29180965 PMC5693842

[19] Rios D. , Boteon A. P. , and Di Leone C. C. L. , et al.Vitamin E: A Potential Preventive Approach Against Dental Erosion—An In Vitro Short-Term Erosive Study, Journal of Dentistry. (2021) 113, 10.1016/j.jdent.2021.103781, 103781. 34400251

[20] Whang H.-J. and Shin D.-H. , Effects of Applying Antioxidants on Bond Strength of Bleached Bovine Dentin, Restorative Dentistry & Endodontics. (2015) 40, no. 1, 37–43, 10.5395/rde.2015.40.1.37. 25671211 PMC4320275

[21] Hobson R. , Vitamin E and Wound Healing: An Evidence-Based Review, International Wound Journal. (2016) 13, no. 3, 331–335, 10.1111/iwj.12295. 25124164 PMC7949595

[22] Kedjarune U. , Charoenworaluk N. , and Koontongkaew S. , Release of Methyl Methacrylate From Heat-Cured and Autopolymerized Resins: Cytotoxicity Testing Related to Residual Monomer, Australian Dental Journal. (1999) 44, no. 1, 25–30, 10.1111/j.1834-7819.1999.tb00532.x. 10217017

[23] Burton G. W. and Traber M. G. , Vitamin E: Antioxidant Activity, Biokinetics, and Bioavailability, Annual Review of Nutrition. (1990) 10, no. 1, 357–382, 10.1146/annurev.nu.10.070190.002041. 2200468

[24] Traber M. G. and Atkinson J. , Vitamin E, Antioxidant and Nothing More, Free Radical Biology and Medicine. (2007) 43, no. 1, 4–15, 10.1016/j.freeradbiomed.2007.03.024. 17561088 PMC2040110

[25] Huang F. M. , Tai K. W. , Hu C. C. , and Chang Y. C. , Cytotoxic Effects of Denture Base Materials on Oral Epithelial Cells and Fibroblasts, International Journal of Prosthodontics. (2001) 14, no. 5, 439–443. 12066639

[26] Rose E. C. , Bumann J. , Jonas I. E. , and Kappert H. F. , Biological Assessment of Orthodontic Acrylic Materials, Journal of Orofacial Orthopedics/Fortschritte der Kieferorthopädie. (2000) 61, no. 4, 246–257, 10.1007/s000560050010. 10961050

[27] Gallorini M. , Petzel C. , and Bolay C. , et al.Activation of the Nrf2-Regulated Antioxidant Cell Response Inhibits HEMA-Induced Oxidative Stress and Supports Cell Viability, Biomaterials. (2015) 56, 114–128, 10.1016/j.biomaterials.2015.03.047. 25934285

[28] Schweikl H. , Spagnuolo G. , and Schmalz G. , Genetic and Cellular Toxicology of Dental Resin Monomers, Journal of Dental Research. (2006) 85, no. 10, 870–877, 10.1177/154405910608501001. 16998124

[29] Ferracane J. L. , Elution of Leachable Components From Composites, Journal of Oral Rehabilitation. (1994) 21, no. 4, 441–452, 10.1111/j.1365-2842.1994.tb01158.x. 7965355

[30] Walther U. I. , Siagian I. I. , Walther S. C. , Reichl F. X. , and Hickel R. , Antioxidative Vitamins Decrease Cytotoxicity of HEMA and TEGDMA in Cultured Cell Lines, Archives of Oral Biology. (2004) 49, no. 2, 125–131, 10.1016/j.archoralbio.2003.08.008. 14693206

[31] Baldion P. A. , Velandia-Romero M. L. , and Castellanos J. E. , Dental Resin Monomers Induce Early and Potent Oxidative Damage on Human Odontoblast-Like Cells, Chemico-Biological Interactions. (2021) 333, 10.1016/j.cbi.2020.109336, 109336. 33248029

[32] Pisoschi A. M. , Pop A. , Iordache F. , Stanca L. , Predoi G. , and Serban A. I. , Oxidative Stress Mitigation by Antioxidants, European Journal of Medicinal Chemistry. (2021) 209, 10.1016/j.ejmech.2020.112891, 112891. 33032084

[33] Dahl J. E. , Frangou-Polyzois M. J. , and Polyzois G. L. , *In Vitro* Biocompatibility of Denture Relining Materials, Gerodontology. (2006) 23, no. 1, 17–22, 10.1111/j.1741-2358.2006.00103.x. 16433637

[34] Sun L. , Ye X. , Ding D. , and Kai L. , Opposite Effects of Vitamin C and Vitamin E on Antifungal Activity, Journal of Microbiology and Biotechnology. (2019) 29, no. 4, 538–547, 10.4014/jmb.1901.01012. 30939634

[35] Divyadharsini V. , Uma Maheswari T. N. , and Rajeshkumar S. , Assessment of Antimicrobial Activity of Lycopene, Vitamin E, and Lycopene-Vitamin E Combination Against *Staphylococcus aureus*, *Streptococcus mutans*, *Enterococcus faecalis*, and *Candida albicans*: An In Vitro Study, Cureus. (2023) 15, no. 7, 10.7759/cureus.42419. PMC10448004 37637570

[36] Naguib M. M. and Valvano M. A. , Vitamin E Increases Antimicrobial Sensitivity, mSphere. (2018) 3, no. 6, 10.1128/mSphere.00564-18. PMC6291622 30541778

[37] Abdallah R. M. and Aref N. S. , Curcumin-Containing Soft Liner for Oral Candidiasis, World Journal of Dentistry. (2021) 12, no. 6, 435–440, 10.5005/jp-journals-10015-1867.

[38] Halliwell B. , Antioxidants and Human Disease: A General Introduction, Nutrition Reviews. (1997) 55, no. 1, S44–S49, 10.1111/j.1753-4887.1997.tb06100.x. 9155225

[39] Naka O. and Tasopoulos T. , et al.Effectiveness of Antimicrobial Agents Incorporated Into Soft Denture Liners: A Systematic Review, Materials. (2025) 18, no. 8, 10.3390/ma18081764, 1764. 40333421 PMC12028984

[40] Godil A. Z. , Bhagat D. , Das P. , Kazi A. I. , Dugal R. , and Satpute S. , Incorporation of Fluconazole and *Ocimum sanctum* Oil in Soft Denture Liners to Treat Biofilms of *Candida albicans* Associated With Denture Stomatitis, Dentistry 3000. (2021) 9, no. 1, 11–22, 10.5195/d3000.2021.120.

[41] Mainieri V. C. , Beck J. , Oshima H. M. , Hirakata L. M. , and Shinkai R. S. , Surface Changes in Denture Soft Liners, Gerodontology. (2011) 28, no. 2, 146–151, 10.1111/j.1741-2358.2010.00375.x. 21054504

[42] Salawi A. and Nazzal S. , The Physiochemical, Mechanical, and Adhesive Properties of Solvent-Cast Vitamin E/Soluplus Films, International Journal of Pharmaceutics. (2018) 552, no. 1-2, 378–387, 10.1016/j.ijpharm.2018.10.018. 30308273

[43] Aref N. S. , Sesame Oil (*Sesamum indicum* L.) as a New Challenge for Reinforcement of Conventional Glass Ionomer Cement, Could It Be?, International Journal of Dentistry. (2021) 2021, 10.1155/2021/5516517, 5516517. 33824660 PMC8007341

[44] Marghalani H. Y. , Effect of Filler Particles on Surface Roughness of Experimental Composite Series, Journal of Applied Oral Science. (2010) 18, no. 1, 59–67, 10.1590/S1678-77572010000100011. 20379683 PMC5349034

